# A photochemical diode artificial photosynthesis system for unassisted high efficiency overall pure water splitting

**DOI:** 10.1038/s41467-018-04067-1

**Published:** 2018-04-27

**Authors:** Faqrul A. Chowdhury, Michel L. Trudeau, Hong Guo, Zetian Mi

**Affiliations:** 1 Department of Electrical and Computer Engineering, McGill University, 3480 University Street, Montreal, QC H3A 0E9 Canada; 2 Center of Excellence in Transportation Electrification and Energy Storage (CETEES), Hydro-Québec 1800 Boul. Lionel-Boulet, Varennes, QC J3X 1S1 Canada; 3 Department of Physics, McGill University, 3600 University Street, Montreal, QC H3A 2T8 Canada; 4 Department of Electrical Engineering and Computer Science, University of Michigan, Ann Arbor, MI 48109 USA

## Abstract

The conversion of solar energy into chemical fuels can potentially address many of the energy and environment related challenges we face today. In this study, we have demonstrated a photochemical diode artificial photosynthesis system that can enable efficient, unassisted overall pure water splitting without using any sacrificial reagent. By precisely controlling charge carrier flow at the nanoscale, the wafer-level photochemical diode arrays exhibited solar-to-hydrogen efficiency ~3.3% in neutral (pH ~ 7.0) overall water splitting reaction. In part of the visible spectrum (400–485 nm), the energy conversion efficiency and apparent quantum yield reaches ~8.75% and ~20%, respectively, which are the highest values ever reported for one-step visible-light driven photocatalytic overall pure water splitting. The effective manipulation and control of charge carrier flow in nanostructured photocatalysts provides critical insight in achieving high efficiency artificial photosynthesis, including the efficient and selective reduction of CO_2_ to hydrocarbon fuels.

## Abstract (web summary)

A major challenge facing solar-to-fuel technologies is the integration of light-absorbing and catalytic components into efficient water-splitting devices. Here, the authors construct a photochemical diode array to harvest visible light and split pure water at high solar-to-hydrogen efficiencies.

## Introduction

Solar water splitting, by harvesting abundant solar energy and storing it in the clean chemical energy form^1–4^, has been considered as one of the most promising approaches of renewable energy production and environmental remediation^5–8^. Significantly, the capacity to directly split seawater is ideally suited for large scale solar fuel production. While tremendous progress has been made in photoelectrochemical (PEC) water splitting in the past decades, it is not suited to split nearly pH neutral water such as sea water. Alternatively, photochemical or photocatalytic dissociation of water has been intensively studied, which can perform one-step overall neutral water splitting. In this approach, the counter electrode, in the form of micro/nano-scale co-catalyst, is monolithically integrated on the photocatalyst surface^9–11^. As such, it has often been referred to as a wireless version of PEC water splitting, which does not require conductive electrolyte and conductive substrate for its operation, as the ionic diffusion problem is greatly reduced due to integrated nature of the device. Owing to the much simpler configuration, photocatalytic water splitting is amenable to cheap, large-scale hydrogen generation^12–16^. However, one inherent limitation with this approach is the simultaneous production of hydrogen (H_2_) and oxygen (O_2_) gases in proximity to each other, which need to be separated safely for practical applications, such as in a Fuel cell.

Both photocatalytic and PEC water splitting approaches intrinsically require four photons, i.e., four active electron–hole pairs to split two water molecules (H_2_O) into one O_2_ and two H_2_ molecules, described as, $2{\mathrm{H}}_2{\mathrm{O}} = 2{\mathrm{H}}_2 + {\mathrm{O}}_2$ (Supplementary Note 1). However, to achieve unassisted overall water splitting using the single-absorber photocatalytic process, the conduction and valence band edges of the photocatalyst must straddle the proton reduction and water oxidation (redox) potentials, respectively, while possessing a sufficiently narrow bandgap to absorb a large part of the solar spectrum. Due to such stringent requirements, there are very few photocatalysts that can perform unassisted, overall water splitting reaction under visible light irradiation. In addition, for semiconductor-based photocatalysts, efficient separation of photo-generated charge carriers (electrons and holes) towards the appropriate catalytic sites has remained challenging, particularly for photocatalytic water splitting wherein no external bias is applied. While numerous efforts have been undertaken to address the critical issue of efficient charge separation through surface and interface engineering and the selective loading of co-catalysts to create spatially separated redox reaction sites^11,17–22^, it has remained elusive to achieve a precise control of charge carrier flow within the bulk and to steer photo-excited electrons and holes to their reduction and oxidation sites, respectively. To date, the best reported solar-to-hydrogen (STH) conversion efficiency for pH neutral photocatalytic water splitting is limited to ~1%, or less, compared to the 10–16% reported for PEC devices^13,16,23–29^ in conductive electrolytes. Recently, it has been discovered that the energy bandgap of group III-nitride semiconductors, e.g., Ga(In)N, can straddle water redox potentials for a wide absorption wavelength range (ultra-violet, visible, and near-infrared)^30–32^, thereby holding an enormous promise for high efficiency one-step overall water splitting^33–36^. Moreover, the surfaces of III-nitride semiconductors can be tuned to be nitrogen-rich to protect against photo-corrosion and oxidation^37,38^, thus making them suitable for stable and efficient photocatalysis.

In this work, we propose and demonstrate multi-band InGaN nanosheet photochemical diode (PCD)^39^ structures, which can spontaneously induce charge carrier separation and steer charge carriers toward the distinct redox sites for water oxidation and proton reduction. During the synthesis of InGaN photochemical diode nanosheet structure, p-type dopant (Mg) concentrations are rationally tailored, which induces a large built-in electric field between the two parallel surfaces. Consequently, the two surfaces are enriched with photo-generated holes and electrons to perform water oxidation and proton reduction reactions, respectively. In addition to the efficient charge carrier separation and extraction, the spatial separation of catalytic sites in such a nanoscale photochemical diode effectively reduces carrier recombination and back reaction. Subsequently, we demonstrate herein a double-band InGaN nanosheet device, which exhibits a solar-to-hydrogen conversion efficiency of ~3.3% for pH neutral overall water splitting. The capacity to achieve controllable charge carrier separation and extraction at the nanoscale will also be instrumental to break the efficiency bottleneck for artificial photosynthesis, including reduction of CO_2_ to hydrocarbon fuels.

## Results

### Design and properties of the photochemical diode

**Fig. 1:**
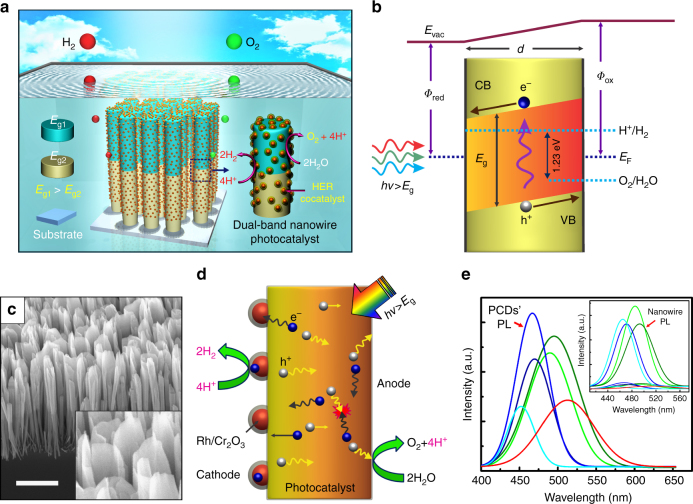
Structural and optical properties of InGaN photochemical diode. **a** Schematic illustration of wafer-level unassisted photocatalytic overall water splitting on double-band nanowire arrays^36^, which are vertically aligned on a planar substrate and decorated with co-catalysts for hydrogen evolution reaction (HER). Unlike tandem PEC cells or photovoltaic (PV) devices^66–69^ this approach does not require any carrier recombination/transfer or current matching between the layers along vertical direction. Both water oxidation and proton reduction reaction occur on the radial non-polar surfaces of each layer. **b** Energy-band representation of the proposed photochemical diode (PCD) with radial thicknes “*d*” showing the built-in electric field (band-bending) that separates the charge carriers (electron and hole) and drives towards the opposite cathode and anode surfaces. In contrast to conventional p-n PCD (Supplementary Fig. 1 and Supplementary Note 1), only single photon absorption is required to generate one active electron–hole pair to participate in redox reaction (like Schottky-type photochemical diode). **c** A 45° tilted SEM image of InGaN:Mg PCD nanostructures, vertically aligned on Si substrate. Scale bar, 1 µm. The magnified image of the nanosheets is also presented in the inset for clarity. **d** Schematic (real space) depiction of the dynamic behaviors of charge carriers in a single-photon PCD upon photoexcitation. Electron enriched surface (cathode) of the PCD is largely decorated with photo-deposited HER co-catalysts (Rh/Cr_2_O_3_ core/shell nanoparticles). **e** Room temperature photoluminescence (PL) spectrum from as-grown p-InGaN PCDs for different indium incorporations (correspond to different bandgaps, depicted using distinct colors). The inset shows ~20-fold reduction in PL intensity for the photochemical diodes compared to that of nanowires

Schematically shown in Fig. 1a is the typical overall neutral pH water splitting on multi-band (GaN/InGaN) nanowire photocatalysts vertically aligned on the substrate (Si). Redox sites (and co-catalysts) in such axially symmetric nanostructures are randomly distributed on the surfaces. In contrast, gradient in p-type dopant (Mg) concentrations leads to a large work function difference (up to 300 meV) between the two parallel surfaces of a photochemical diode. The resulting p-p^+^ nanoscale lateral junction, schematically illustrated in Fig. 1b, induces unidirectional flow of photo-excited charge carriers, i.e., electrons and holes migrate toward the surfaces with a relatively small and large work function (*Φ*_red_ and *Φ*_ox_), respectively. Shown in Fig. 1a, the energy bandgap of the nanosheet structures can be further varied along the vertical direction, i.e. the photon absorption path. The resulting multi-band photocatalysts promise photocatalytic solar water splitting with the highest efficiency possible^7,40^. Figure 1b schematically illustrates the energy bands of the proposed InGaN nanosheet structures, which are grown directly on Si substrate using plasma-assisted molecular beam epitaxy (MBE) . During the epitaxy process, p-type dopants (Mg) are impingent primarily on one side of the nanosheet structure (see Methods). The resulting Mg-doping gradient along the lateral dimension of the nanosheet establishes a strong built-in electric field, schematically shown in Fig. 1b, which separates the photo-generated electrons and holes, and drives them towards opposite surfaces, thus reducing the probability of recombination. Figure 1c shows the typical bird’s-eye-view SEM images of InGaN nanostructures, which are comprised of axially asymmetric nanosheets with parallel non-polar surfaces. A simplistic view of the dynamic behaviors of charge carriers are depicted in Fig. 1d, which includes electron–hole pair generation upon photo-excitation, bulk recombination, carrier separation and migration towards laterally opposite direction. Consequently, the two catalytic surfaces are enriched with electrons and holes, respectively. The electron enriched surface (cathode) of the photochemical diode largely facilitates photo-deposition of proton reduction co-catalysts (Rh/Cr_2_O_3_ core/shell nanoparticles) (see Methods), which in turn enhances the hydrogen evolution reaction (HER) significantly. Water oxidation reaction takes place on the hole enriched surface (anode). Details about conventional photochemical diode and junction engineering approach can be found in Supplementary Fig. 1 and Supplementary Note 1. One direct evidence for the efficient charge carrier separation and extraction of the presented InGaN nanosheet structures, compared to the conventional nanowires, is the significantly reduced photoluminescence intensity. Shown in the inset of Fig. 1e, the photoluminescence (PL) emission intensity of InGaN nanosheets is nearly 20 times smaller, compared to that of InGaN nanowires grown under similar conditions. Therefore, with significantly reduced charge carrier recombination, InGaN nanosheets are expected to exhibit noticeably higher photocatalytic activity than corresponding nanowire structures^41^ (Supplementary Figs. 2–3 and Supplementary Notes 2–3). By varying the epitaxy conditions, the energy bandgap of InGaN nanosheets, evident by the photoluminescence emission spectra, can be tuned over a large part of the visible spectral range, shown in Fig. 1e.

### Surface selectivity for oxidation and reduction

**Fig. 2:**
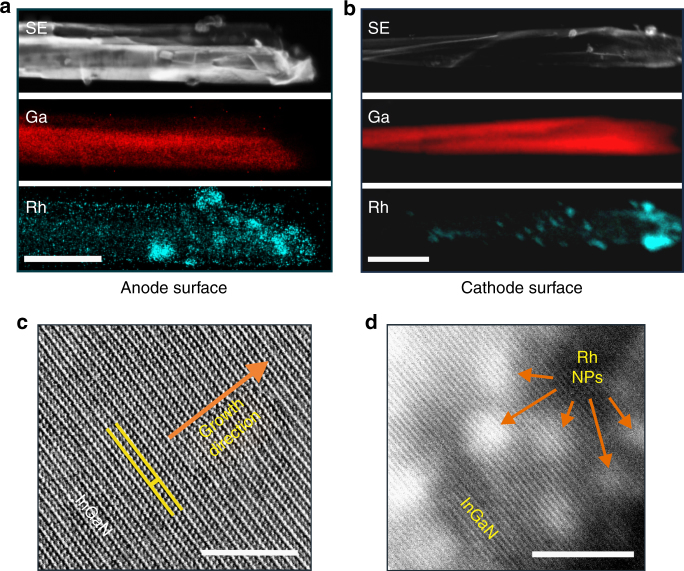
Surface selectivity of InGaN photochemical diode for Rh-nanoparticle deposition. Comparison of STEM-SE and EDXS elemental mapping on two different surfaces of InGaN nanosheet (decorated with Rh-nanoparticles) shows that **a** very few Rh nanoparticles were deposited on the anode (outer) surface, whereas **b** significantly large number of Rh-nanoparticles get deposited on the cathode (inner) surface. Scale bars, 400 nm. HRSTEM-BF lattice fringe image from InGaN photochemical diode nanosheet, illustrating **c** defect-free single crystalline In_0.22_Ga_0.78_N anode surface, and **d** Rh nanoparticles on the crystalline cathode surface of photochemical diode. Scale bars, 5 nm. A radial density filter was used for Fig. 2c

Scanning transmission electron microscopy (STEM) imaging, supported by the energy dispersive X-ray scanning (EDXS) analysis on p-type InGaN nanosheet photochemical diodes, decorated with Rh-nanoparticles, shows a significant difference in the number of nanoparticles loading between the two parallel surfaces. As shown in Fig. 2b, preferential photo-reduction of Rh-metal precursors to Rh-nanoparticles is facilitated on the reduction surface (cathode) due to its electron enrichment compared to that on the oxidation surface (anode) in Fig. 2a. Scanning transmission electron microscopy (STEM)-ZC/BF images further confirm Rh-nanoparticles’ deposition on the reduction sites of InGaN photochemical diode nanostructure (Supplementary Fig. 4). High-resolution STEM bright-field lattice image also depicts high crystalline quality of the defect-free In_0.22_Ga_0.78_N nanostructure surfaces, shown in Fig. 2c, d. For comparison, non-selective, rather random distribution of metal nanoparticles on the non-polar surfaces of conventional nanowires are also presented in Supplementary Fig. 5.

**Fig. 3:**
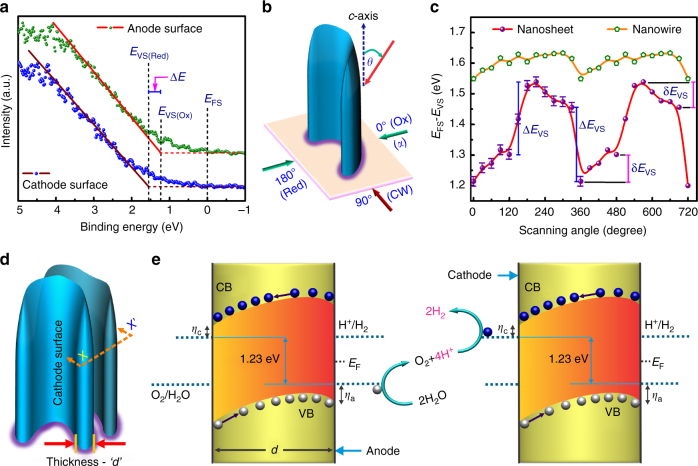
Surface charge properties of In_0.22_Ga_0.78_N:Mg photochemical diode. **a** ARXPS valence spectrum for cathode and anode surface of p-InGaN photochemical diode nanosheets, depicting the offset in surface valence band maximum (*E*_VS_) relative to surface Fermi-level (*E*_FS_). **b** Schematic illustration of probing photochemical diode surfaces for valence spectra using ARXPS. Angles on the imaginary plane normal to *c*-axis (parallel to the substrate) are the radial scanning angles (α, clockwise), and “*θ’*“ denotes the angle of X-ray excitation relative to c-axis (See Methods). **c** *E*_FS_−*E*_VS_ for Mg-doped In_0.22_Ga_0.78_N nanosheets and nanowire arrays, derived from ARXPS valence spectrum as a function of scanning angle. Periodic fluctuation is clearly observed for *E*_FS_ position on the photochemical diode nanosheets relative to *E*_VS_. An error bar of ~±0.03 eV corresponds to uncertainties involved in measuring *E*_VS_ and C 1s peak. **d**, 3D depiction of photochemical diode nanosheets with an arbitrary radial thickness *‘d’*. Inner surface of the curved nanosheet is denoted as the cathode surface as per Fig. 2. **e** Neutral pH overall water splitting on the surfaces of photochemical diode nanostructures, presented schematically as a top view at the plane (X-X′) of cross-section in Fig. 3d. *η*_a_ and *η*_c_ represents the anodic and cathodic over-potentials for water oxidation and proton reduction reaction, respectively. With the directional (opposite) migration of electrons and holes, redox reactions can be coupled between parallel (cathode and anode) surfaces of vertically aligned adjacent photochemical diode nanosheets

To further gain a deep insight regarding the deviation in photo-deposition behavior, near-surface band-structure of as-grown p-InGaN nanowires and p-InGaN nanosheets were characterized using angle resolved X-ray photoelectron spectroscopy (ARXPS). Illustrated in Fig. 3a, the measured surface valence band maximum (*E*_VS_) values between the two non-polar surfaces (relative to surface Fermi-level, *E*_FS_) are significantly different, with *E*_VS_ for the cathode surface being ~300 meV larger than that for the anode surface. This suggests the presence of a built-in potential ~300 meV (Δ*E*) along the lateral dimension of the nanosheet structure, as shown schematically in Fig. 1b. Subsequently, surface dependence of *E*_VS_ was analyzed by measuring the valence spectra vs. radial scanning angle, α (Fig. 3b). Variations of *E*_FS_−*E*_VS_ as a functional of scanning angle is illustrated in Fig. 3c for the entire range of α, further confirming the strong dependence of *E*_VS_ on different surfaces. The sharp change in *E*_FS_-*E*_VS_ (Δ*E*_VS_) vs. scanning angle can be ascribed to the transition from one parallel surface to another, e.g., from anode to cathode surface, whereas the slow and gradual change (δ*E*_VS_) is attributed to the curvature and orientation of nanosheet arrays. In contrast, conventional InGaN nanowires exhibit nearly constant *E*_VS_ at different scanning angles, also shown in Fig. 3c for comparison. Further comparative analysis between the surface potentials of nanowire and nanosheet structures is discussed in Supplementary Fig. 2 and Supplementary Note 3. A quantitative estimation for the band-diagram of InGaN nanosheet structures is shown in Supplementary Fig. 6a, which is derived from the XPS and TEM analysis performed on InGaN nanosheets with optimum bandgap for enhanced photocatalytic activity. It is evident that the large built-in potential leads to the spontaneous accumulation of electrons and holes on the cathode and anode surfaces, respectively^36^. This implies that the origin of preferential photo-deposition of noble metal nanoparticles on cathode surface of p-InGaN photochemical diode, as shown in Fig. 2a–d, is due to the reduction of noble metal precursors by photo-excited electrons enriched on that surface.

It is worthwhile mentioning that anisotropic facet-dependent co-catalyst deposition had been reported previously to ensure spatial separation of oxygen evolution reaction (OER) and HER co-catalysts^42–48^, and thus to provide enhanced carrier separation in the near-surface region (Supplementary Note 4). However, bulk recombination remains a limiting factor for their low apparent quantum efficiency in water splitting. Unique to the presented photochemical diode nanostructure is the net lateral band-bending between two spatially separated redox surfaces. Water oxidation and proton reduction reactions occur at the two distinct reaction sites on photochemical diode nanosheets, and are coupled between the parallel anode and cathode surfaces^49,50^, schematically illustrated in Fig. 3d, e. Under concentrated sunlight, the band-bending can be reduced in the bulk, which can further lower the recombination probability by making the built-in electric field nearly linear and hence the flow/separation of charge carriers unidirectional (Supplementary Fig. 6b).

### Characterization and performance analysis of double-band PCD

**Fig. 4:**
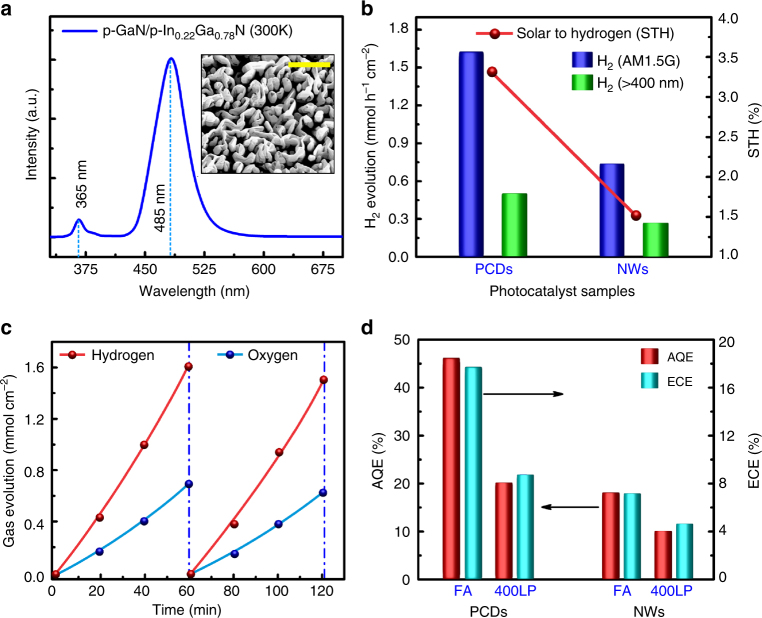
Enhanced STH efficiencies on double-band GaN:Mg/InGaN:Mg photochemical diodes. **a** Room temperature photoluminescence (PL) spectrum depicting optical emission peaks at ~365 nm (GaN) and at ~485 nm (In_0.22_Ga_0.78_N). The inset shows 15° tilted SEM image of the photochemical diodes. Scale bar, 1 µm. **b** H_2_ evolution rate in overall neutral (pH ~ 7.0) water splitting for various photocatalyst samples under different excitation conditions. All the photocatalysts contain Rh/Cr_2_O_3_ as HER co-catalyst, photo-deposited on the surface. Photochemical diodes provided two-fold enhancement in solar to hydrogen (STH) conversion efficiency compared to their nanowire counterparts. **c** Stoichiometric H_2_ and O_2_ evolution rate and the time course of overall water splitting, demonstrating balanced redox reaction and stability of nanowire photochemical diodes. **d** Comparative illustration of apparent quantum efficiency (AQE) and energy conversion efficiency (ECE) for different photocatalyst samples, derived under full arc using AM1.5 G filter (FA) and 400 nm long-pass filter (400LP)

Double-band GaN:Mg/InGaN:Mg nanostructures were grown on Si wafer using plasma-assisted molecular beam epitaxy (See Methods)^34–36^. The nanosheet structures are vertically standing on the Si substrate, having an areal density in the range of ~1.5 × 10^10^ cm^−2^ (Fig. 1c). The photochemical diodes have an average height ~1.5–2 µm, and the thickness varies from ~50 to 120 nm. The PL spectra revealed an optical emission peak at ~485 nm, as shown in Fig. 4a, which can be attributed to InGaN bandgap of 2.56 eV. The average indium incorporation is estimated to be ~22% for the grown nanostructures. Detailed STEM and EDXS analysis confirms the existence of a continuous long InGaN segment, simultaneously showing the distribution of Rh/Cr_2_O_3_ nanoparticles on the surface^34,36^ (Supplementary Note 5). The p-type behavior of Mg-doped crystalline In_0.22_Ga_0.78_N photochemical diodes is confirmed by photo-electrochemical characterization that includes open-circuit potential (OCP), Mott-Schottky and photocurrent measurements (Supplementary Fig. 7 and Supplementary Note 6).

The nanosheet arrays were tested for both hydrogen evolution reaction (HER) in aqueous methanol (CH_3_OH) solution, as well as neutral pH overall water splitting (OWS). A 300 W Xenon lamp was used as a concentrated irradiation source for photo-excitation, which has an intensity equivalent to ~32 suns when measured on the nanostructure substrate (Supplementary Fig. 8). Rh nanoparticles and Rh/Cr_2_O_3_ core–shell nanostructures were photo-deposited as the co-catalysts for HER and OWS reactions, respectively. In the wavelength range of 200–485 nm (incident intensity of ~611 mW cm^−2^, see Supplementary Notes 7–8), stoichiometric gas evolution from neutral pH water splitting was measured at a rate of ~1.62 mmol h^−1^ cm^−2^ H_2_ and ~0.784 mmol h^−1^ cm^−2^ O_2_, resulting in an AQE ~45.85%, which is more than two-fold higher than previously reported AQE of ~20% for double-band nanowire heterostructures^36^. Time evolution of photocatalytic hydrogen production from the photochemical diode arrays are shown in Supplementary Fig. 9. Under visible light irradiation (> 400 nm), the evolution rate was measured as ~0.5 mmol h^−1^ cm^−2^ for H_2_, and the AQE from the photochemical diode nanosheets (nanowires) was estimated to be ~19.93% (12.3%).

Evidently, significant enhancement in overall photocatalytic water splitting activity had been derived from photochemical diode nanosheets compared to that from nanowire heterostructures. For comparison, the amount of hydrogen evolution from the photochemical diodes is increased by more than a factor of two using full arc illumination with AM1.5 G filter. This, in turn, enhanced the energy conversion efficiency (ECE) from ~7.5 to ~17.5%. Moreover, an impressive ~3.3% of solar-to-hydrogen conversion efficiency has been measured in this study, which is significantly higher than that estimated from dual-band nanowire structures, as depicted in Fig. 4b. Repeated cycles for the stoichiometric hydrogen and oxygen evolution in neutral pH water splitting using AM1.5 G filter are demonstrated in Fig. 4c (Supplementary Movie 1 and 2). Illustrated in Fig. 4b, d are the comparative study of hydrogen evolution and corresponding AQE and ECE from neutral pH overall water splitting under full arc illumination using AM1.5 G optical filter and under visible light irradiation using a 400 nm long-pass optical filter. The photochemical diode nanostructures remain stable after the photocatalytic reactions, and negligible signs of degradation was observed after ~4 h of overall neutral pH water splitting and hydrogen evolution reaction from aqueous methanol solution (shown in Supplementary Fig. 10). The stability of the co-catalyst nanoparticles on photocatalyst surface was further confirmed from TEM analysis.

## Discussion

In the end, we discuss the unique charge transfer mechanism in nanosheet photochemical diodes and the impact on solar-to-hydrogen efficiency in photocatalytic overall water splitting. In nanostructured photocatalysts, charge carrier transport is no longer diffusion limited; the effective extraction of photo-generated charge carrier is often restricted by surface and interface electronic properties, e.g., the presence of surface band bending^34,36,51–53^. To date, it has remained a grand challenge to precisely steer charge carrier flow in nanostructured photocatalysts, due to the lack of control over their surface band bending. In our previous studies, we have demonstrated that by minimizing the surface potential for hole transport, the STH efficiency for photocatalytic overall pure water splitting on InGaN nanowire arrays was significantly enhanced from <0.1 to over 1%^34–36^. Further improvement of the STH efficiency, however, has been fundamentally limited by charge carrier recombination (Supplementary Fig. 11a-b), including both surface and bulk recombination. In this work, we have shown that such a critical challenge can be effectively addressed in nanosheet photochemical diodes. The asymmetric dopant incorporation in InGaN nanosheets and the resulting built-in electric field, schematically shown in Fig. 1b, offers several essential benefits for photocatalytic overall water splitting. It leads to the unidirectional charge carrier flow, i.e., the accumulation of photo-generated electrons and holes on the cathode and anode surfaces of the same nanosheet structure, respectively. The efficient separation of photo-excited charge carriers is unambiguously supported by the preferential deposition of HER co-catalysts only on the cathode surfaces of InGaN nanosheets (Fig. 2b), the significant reduction of photoluminescence emission compared to conventional nanowires (inset of Fig. 1e), and the large difference in surface potential (~300 meV) between the anode and cathode surfaces (Fig. 3c). Over 90% of the photo-excited electrons and holes are spatially separated on the cathode and anode surfaces^36^, thereby minimizing both surface and bulk recombination^54^, detailed in Supplementary Fig. 11. Moreover, reverse diffusion currents due to the concentration gradient is minimized by the energy barriers (Supplementary Fig. 11c-d, Supplementary Note 9) and surface trapping of carriers in co-catalyst nanoparticles^17,19–22^. Significantly, the rational design and synthesis of anode and cathode surfaces in nanostructured photocatalysts can effectively increase the surface area for water oxidation reaction, which is often the rate-limiting process of water splitting^55–59^. Water oxidation and proton reduction reactions can be coupled between spatially separated reaction sites (parallel electrodes), i.e., the cathode and anode surfaces^49,50^ of two adjacent photochemical diodes (Fig. 3e), thereby drastically suppressing back reaction. In addition, the effective surface relaxation of nanosheet structures allows for the optimization of the bandgap and band-bending of InGaN photochemical diodes through variations in indium incorporation and Mg-doping concentration, to ensure sufficient cathodic and anodic over-potentials, and to minimize the surface potential barrier for achieving high photocatalytic efficiency.

The demonstrated STH ~3.3% is significantly higher than previously reported efficiency values for neutral pH one-step overall water splitting^13,16,23^, which generally range from 0.1 to 1.1%. It is worth mentioning that an STH of ~5% had been reported on CoO nanocrystals^14^, which are barely stable in harsh photocatalytic environment. The nanocrystal surfaces become corroded due to difficulties in co-catalyst loading, and further studies seem to be necessary to understand the mechanism and to confirm the reproducibility^16^. Recent studies on a different 2-step approach of water splitting^15^ showed ~2% of STH using CDots-C_3_N_4_. This approach requires efficient generation as well as subsequent decomposition of H_2_O_2_, putting further constraints on the bandgap of suitable photocatalyst (>1.78 eV, compared to ~1.23 eV for neutral pH overall water splitting, excluding necessary over-potentials). A device comprised of catalysts loaded on triple-junction photovoltaic cell demonstrated STH of ~2.5% in an earlier study^60^, which, however, utilizes conductive 1 M potassium borate electrolyte (pH ~ 9.2). Noticeably, many of these relatively high efficiency devices^16,60^ were designed based on conventional photochemical diode^61^ (Supplementary Note 1) which requires the use of ohmic-contact for efficient charge carrier transport. For example, co-catalyst loaded SrTiO_3_:La,Rh|Au(contact)|BiVO_4_:Mo device^16^ increases the STH up to ~1.1% compared to that of ~0.1% using their powder suspensions in Z-scheme with Fe^3+/2+^ redox couples^62^. The use of planar ohmic-contact reduces active surface area for redox reaction and often requires conductive electrolyte to compensate for the distance between cathode and anode surfaces (by selectively adjusting the pH or adding supporting electrolyte in near-neutral pH condition). Moreover, it has been extremely difficult to realize such ohmic-contact in nanostructured photocatalysts. In this context, our nanosheet photochemical diodes do not require ohmic-contact or majority carrier recombination. They offer large (and hole-enriched) anode surface for water oxidation, while enabling selective deposition of HER co-catalysts (Rh/Cr_2_O_3_) on electron-enriched cathode surface, with the least probability of carrier interference, crowding, and recombination. Moreover, solar energy, being a planar resource, can be more effectively harvested (per unit area) in such vertically aligned wafer-level nanosheet structures.

In summary, we have demonstrated photochemical diode artificial photosynthesis system that can enable relatively efficient overall pure water splitting (STH ~3.3%). The wafer level photochemical diodes consist of vertically aligned InGaN nanosheets, with well-defined anode and cathode surfaces for water oxidation and proton reduction, respectively. Unique to such nanosheet photochemical diodes is that charge carrier flow can be precisely controlled at the nanoscale without any external bias: photo-generated electrons and holes are instantaneously separated due to the built-in electric field along the lateral dimension of nanosheets, leading to the spontaneous population of anode and cathode surfaces by holes and electrons, respectively. The spatially separated electron and hole gas significantly minimizes surface and bulk recombination and suppresses back reaction, which have been some of the major challenges in achieving efficient photocatalytic water splitting to date. The effective manipulation and control of charge carrier flow in nanostructured photocatalysts not only significantly enhances the efficiency of photocatalytic water splitting, but also provides critical insight in achieving high efficiency artificial photosynthesis, including the efficient and selective reduction of CO_2_ to hydrocarbon fuels. Future work also includes the development of an axial photochemical diode by connecting the two semiconductor segments of the nanowire/nanosheet with a transparent tunnel junction or transparent ohmic contact, which can significantly enhance the STH efficiency^63,64^.

## Methods

### Molecular beam epitaxial (MBE) growth

InGaN photochemical diode nanostructures were grown on Si (111) substrate by radio frequency (RF) plasma-assisted MBE under nitrogen rich conditions. To remove organic contaminants, the Si substrate was thoroughly cleaned with acetone and methanol solvent. The native oxide on the substrate was removed prior to loading into the MBE chamber by cleaning with 10% hydrofluoric acid (HF). The residual oxide was then desorbed, by in situ annealing of the substrate at ~780 °C before the growth initiation. The clean Si (111) 7 × 7 reconstructed surface from reflection high-energy electron diffraction (RHEED) analysis further confirms the desorption. To promote the formation and nucleation of nanowires, a thin (~1 ML) Ga seeding layer was deposited. Ga, In, and Mg fluxes were controlled using respective thermal effusion cells, whereas the nitrogen radicals were supplied from an RF-plasma source. The growth conditions were optimized after several iterations for better crystalline quality and photocatalytic performance in overall water splitting reaction. Instead of multi-stack InGaN:Mg/GaN:Mg layers^35,36^, a continuous InGaN:Mg layer was grown spontaneously on top of GaN:Ge nanowire template followed by a final GaN:Mg capping layer (relatively smaller than InGaN in length, in proportion to fraction of UV region in the spectrum). A nitrogen flow rate of 1.0 standard cubic centimeters per minute (sccm), and a forward plasma power of ~350 W were used during the growth. Other growth parameters include Ga beam equivalent pressure (BEP) in the range of 6 × 10^−8^ Torr, In BEP approximately 7.8 × 10^−8^ Torr, Mg BEP in the range of 2 × 10^−11^ Torr corresponding to Mg cell temperature (*T*_Mg_) of ~200 °C, and Mg BEP of ~1.5 × 10^−9^ Torr which corresponds to *T*_Mg_ of ~270 °C. To facilitate the formation of Mg-doping gradient in the lateral direction and to counteract the spinodal decomposition of In-N in the surface, the substrate was kept steady during the growth of the photochemical diode. Amount of nitrogen species and metal fluxes in the growth environment were further optimized to ensure essential nitrogen vapor pressure in the vicinity of the growth front, and to reduce long-distance diffusion of indium atom along the growth axis. Mg-doping gradient (due to the surface dependent incorporation) can also be influenced by the super-saturation for Ga-rich or N-rich condition at the localized level^65^. The GaN template was grown at ~780 °C, and the growth temperatures for InGaN were varied from 650 to 705 °C.

### Micro-photoluminescence (μ-PL)

A 405 nm laser or a 325 nm He-Cd laser (Kimmon Koha) was used as excitation source for the μ-PL measurement. The laser beam was focused to a circular spot (~5 μm) on the sample using a 60× objective, which also collects the emitted light from the sample. The collected optical emission was then spectrally resolved using a high-resolution spectrometer (JY HR-550) and detected by a CCD or a photon counting mode photomultiplier tube.

### Micro-Raman analysis

The room-temperature micro-Raman measurements were performed using an external 514 nm argon ion laser as excitation source. A 50× objective (numerical aperture ∼0.9) was employed for focusing the laser excitation to a spot beam of ∼1 μm diameter and to direct an estimated ∼7 mW of power on the sample surface. The Raman signal was collected in the backscattering geometry where the (non-polarized) laser beam was incident at normal to the planar substrate. The Raman spectra were then resolved via an 1800 l mm^−1^ grating, and detected at 0.2 cm^−1^ resolution by a CCD, mounted on the inVia confocal Raman spectrometer from Renishaw.

### Scanning transmission electron microscopy (STEM)

STEM-SE, STEM-BF, and STEM-HAADF imaging were performed using a Hitachi HD2700 Cs-corrected dedicated STEM, which is equipped with a cold field emission emitter and operated at 200 kV with an electron beam diameter of ~0.1 nm (~0.2 nm in HR mode). A 60 mm^2^ silicon drift detector (SDD) from Bruker was used for STEM EDXS analysis. The image in Fig. 2c was processed using a radial difference filter.

### Photo-deposition of co-catalysts

The photochemical diode and the nanowire arrays were decorated with Rh/Cr_2_O_3_ core–shell nanostructures using a stepwise photodeposition technique from their respective liquid precursors. Rh nanoparticles were photodeposited from 14 µl of sodium hexachlororhodate (III) (0.5 M Na_3_RhCl_6_, Sigma-Aldrich), which is subsequently followed by the Cr_2_O_3_ photodeposition from 14 µl of potassium chromate (0.5 M K_2_CrO_4_, Sigma-Aldrich) precursor in aqueous methanol solution (20%). The stepwise deposition ensures the formation of Rh/Cr_2_O_3_ core–shell nanostructures which prevent the back reaction.

### Angle-resolved X-ray photoelectron spectroscopy (ARXPS)

Thermo-Fisher Scientific K-Alpha XPS system was used for the analysis. The system was equipped with a monochromatic Al-Kα X-ray source (*hυ* = 1486.6 eV) and 180° double focusing hemispherical analyzer. The analysis chamber pressure was as low as 10^−8^ Torr. The non-polar surfaces of the nanostructure arrays were excited with the X-ray beam, incident at 60° angle with substrate normal. The *E*_FS_−*E*_VS_ was estimated from the ARXPS valence spectra. Both Au 4f (84.0 eV) and C 1s (284.8 eV) peaks were used to calibrate the binding energies. As shown in Fig. 3a, the position of surface valence band (*E*_VS_) with respect to the surface Fermi level (*E*_FS_, binding energy = 0 eV) can be estimated by measuring the intersection point between the linear extrapolation of the valence band leading edge and the extended baseline.

### Photocatalytic reaction

The experimental configuration was composed of a reaction system and an evaluation system. The wafer sample, fixed by a homemade PTFE holder was placed in a Pyrex chamber containing appropriate solutions. Prior to each photocatalytic experiment, the distilled water was purged for 20–30 min using Ar gas to remove the dissolved gases. After evacuating the chamber, the system was illuminated by a Xenon lamp (Cermax, PE300BUV); adequate transmittance for both UV and visible light was secured by placing a quartz lid in between the reaction chamber and the lamp. The evolved gases were then collected using a vacuum-tight syringe and evaluated by a gas chromatograph (GC, Shimadzu GC-8A) equipped with a thermal conducting detector (TCD). High purity Ar was used as carrier gas. Wavelength dependence of the photocatalytic activity and quantum efficiency was systematically analyzed and measured by using appropriate high-pass and band-pass filters. Due to the manual sampling of the H_2_ and O_2_ gases, the experimental error was approximately 10%.

### Data availability

The data that support the findings of this study are available within the paper [and its supplementary information files]. Further details regarding the data are available from the corresponding author upon reasonable request.

## Electronic supplementary material

Supplementary Information

Description of Additional Supplementary Information

Supplementary Movie 1

Supplementary Movie 2
