# Supplementary material for: A photochemical diode artificial photosynthesis system for unassisted high efficiency overall pure water splitting
Source: Nat Commun. 2018 Apr 27;9:1707. doi: 10.1038/s41467-018-04067-1 (PMC5923260; doi:10.1038/s41467-018-04067-1)
Supplement: Supplementary file 1 — Supplementary Information [file 41467_2018_4067_MOESM1_ESM.pdf]

A Photochemical Diode Artificial Photosynthesis System for Unassisted High Efficiency Overall Pure Water Splitting

Chowdhury *et al.*

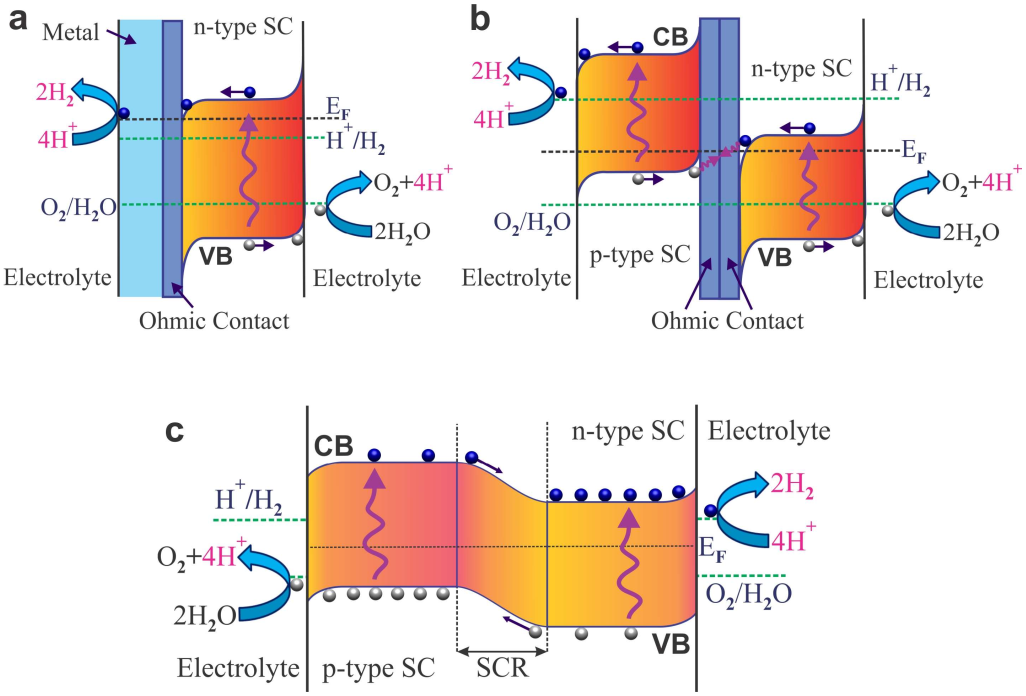

Supplementary Figure 1: Carrier-separation approaches for overall water splitting. a, Schematic illustration of a Schottky-type photochemical diode for overall water splitting, where a single-bandgap n-type semiconductor absorber forms a metal-semiconductor junction via an ohmic contact. Water oxidation occurs on the semiconductor surface and proton reduction occurs on the surface of metal electrode. **b,** Energy-level representation of p-n photochemical diode and redox potentials for overall water splitting, where two semiconductor absorber layers (p-/n-type, assuming identical bandgap) are connected via an ohmic contact as a recombination layer for majority carriers. **c,** Schematic of a p-n homo-junction for overall water splitting. In this majority carrier-device, electrons and holes in the space charge region (SCR) are drifted in opposite direction by the built-in electric field. Suitable co-catalyst and metal contact can be deposited on the surface to facilitate charge carrier extraction and waters splitting reaction.

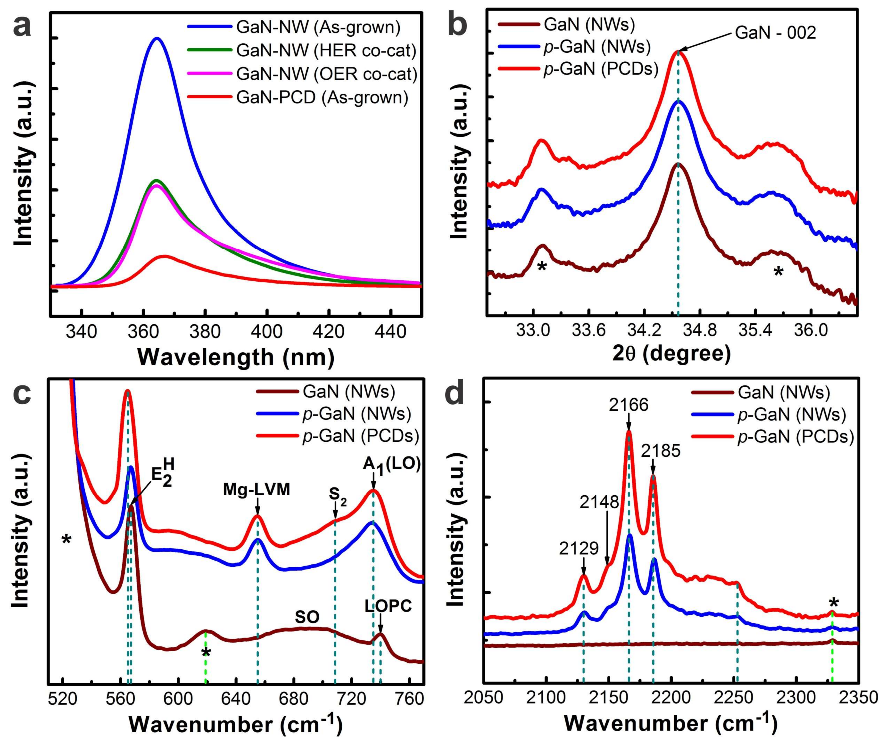

Supplementary Figure 2: Properties of GaN photochemical diodes. a, Room temperature μ-PL spectrum from as-grown and co-catalyst decorated (Rh//Cr_2O_3 for HER and Co_3O_4 for OER) GaN nanowire arrays. The μ-PL spectrum from as-grown GaN photochemical diodes clearly shows ~6-fold reduction in PL intensity compared to that of GaN nanowires. **b,** The prominent reflections of 002 in the XRD pattern for GaN nanowires and photochemical diodes. Log(Intensity) of the spectrum is plotted in a linear scale. Typical micro-Raman spectra of non-doped GaN nanowires and Mg-doped GaN nanowires, along with Mg-doped GaN photochemical diodes **c,** from 510 cm^{-1} to 770 cm^{-1} and **d,** from 2050 cm^{-1} to 2350 cm^{-1}. Mg-cell temperature was optimized to 265 °C for both GaN nanowires and photochemical diodes to provide enhanced photocatalytic activity. The spectra in XRD and micro-Raman are vertically shifted for visual clarity. Signal contributions from the background/substrate are marked with '*' in the respective figures.

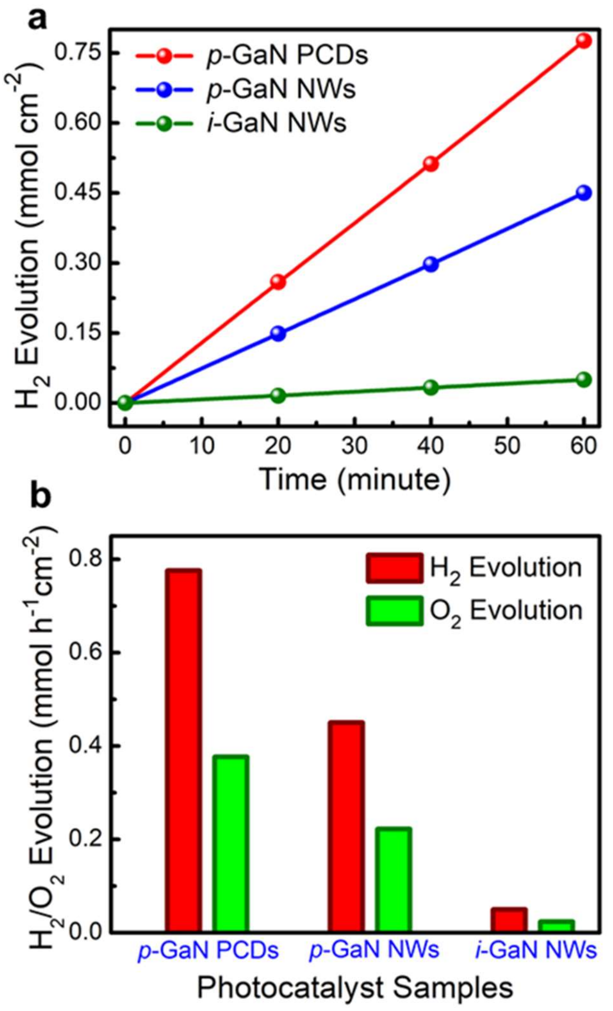

Supplementary Figure 3: Photocatalytic performance of GaN photochemical diodes. a, Time evolution of the amount of hydrogen from neutral pH overall water splitting (OWS) reactions on non-doped GaN nanowires, Mg-doped GaN nanowires and photochemical diodes under concentrated full arc excitation using a 300 W Xenon lamp. Rh/Cr_2O_3 core-shell nanoparticles were photo-deposited as hydrogen evolution reaction (HER) co-catalyst on all the photocatalyst samples prior to the water splitting reaction. **b,** Stoichiometric H_2 and O_2 evolution rate from overall neutral (pH~7.0) water splitting reactions on different photocatalyst samples under concentrated full arc irradiation, depicting 75% enhancement in photocatalytic activity of GaN photochemical diodes compared to that of Mg-doped GaN nanowires.

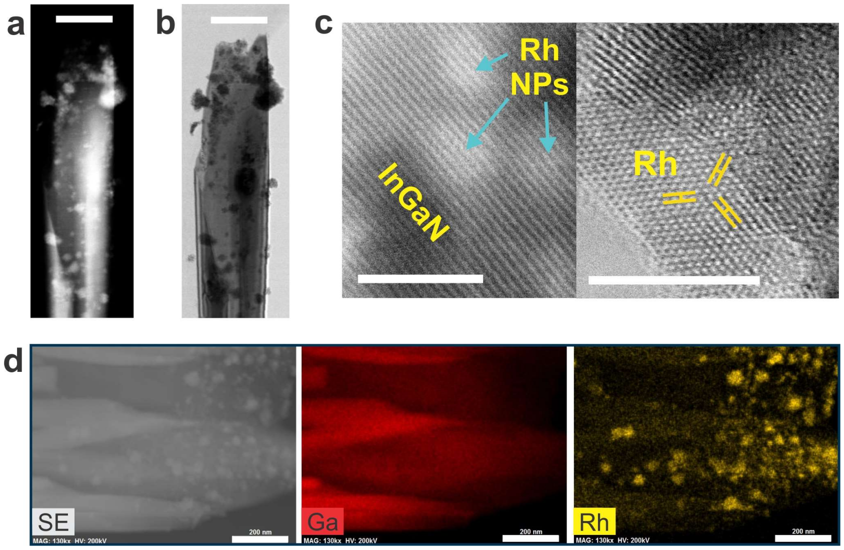

Supplementary Figure 4: Selective deposition of HER co-catalysts on cathode surfaces. STEM-ZC/BF images of Rh-nanoparticles' deposition on the reduction sites (cathode surface) of InGaN photochemical diode nanostructure is shown in **a,** and **b,** respectively. **c,** HRSTEM-BF lattice image of a Rh deposited InGaN photochemical diode surface, which clearly depicts the existence of Rh nanoparticles, and the crystalline quality of Rh cluster. Scale bar, 5 nm. **d,** STEM-SE and EDXS imaging of single InGaN photochemical diode nanosheets (cathode surface), decorated with Rh-nanoparticles. Comparing SE image and EDXS elemental mapping of Rh shows negligible signal from particle deposition on the anode surface (back, outer surface), clearly highlighting the relative difference in particle deposition density. Scale bars, 200 nm.

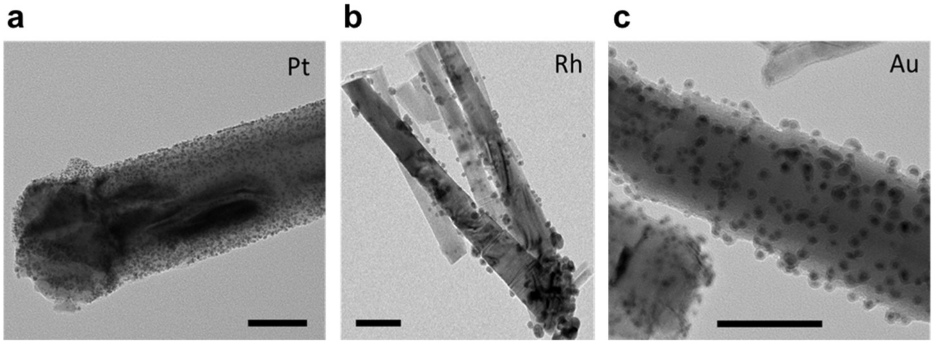

Supplementary Figure 5: Random distribution of HER co-catalysts on nanowire surfaces. Non-selective, rather uniform distribution of noble metals, such as **a,** Pt, **b,** Rh and **c,** Au nanoparticles on the non-polar surface of axially symmetric GaN nanowires. The nanoparticles were photo-deposited using respective precursors. The nanowires as well as the precursor loading were not optimized for enhanced photocatalytic activity. Scale bars, 50 nm.

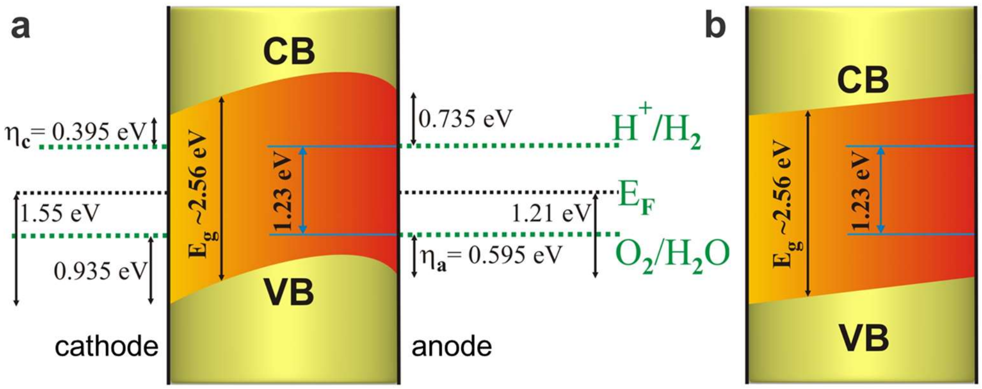

Supplementary Figure 6: Energy bands and over-potentials of InGaN photochemical diode. **a,** A quantitative estimation of the band-diagram of InGaN nanosheet photochemical diode, supported by the XPS and TEM analysis for optimum bandgap, as shown in Fig. 2 and Fig. 3. **b,** Under concentrated sunlight, the band-bending in the bulk can be reduced due to the band-flattening effect. The effect can lower the recombination probability (for both bulk and surface recombination) further due to linear built-in electric field and hence making the flow/separation of the charge carriers unidirectional (opposite), as shown in Fig. 1b.

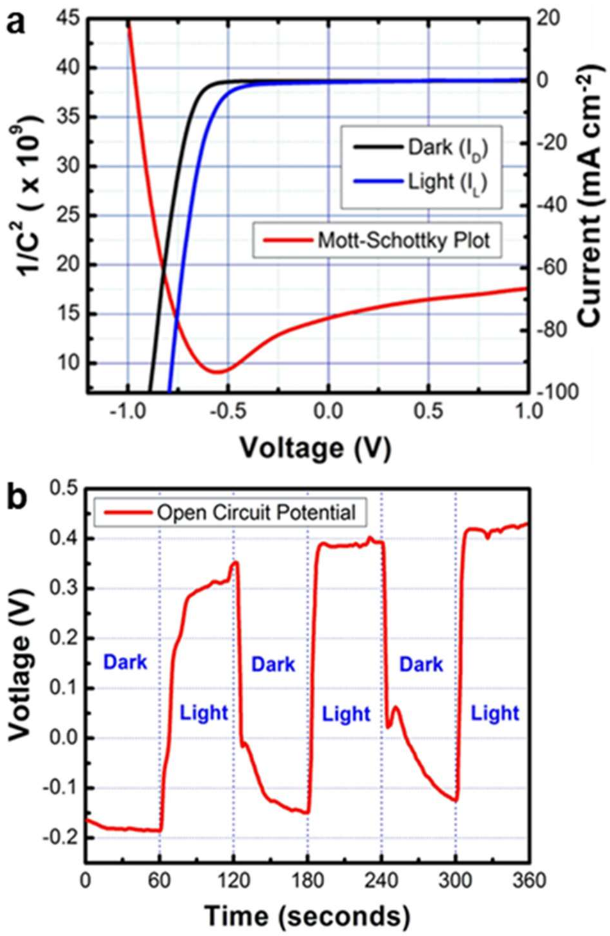

Supplementary Figure 7: Photo-electrochemical characterization of InGaN photochemical diodes. a, Mott-Schottky plot of the *p*-InGaN photochemical diode nanostructure arrays in the dark, and I-V curve of the same sample under light illumination (full arc) and in dark. Note that, with the direct formation of p-InGaN on n-type template/substrate, the sample is not designed for photoelectrochemical water splitting. **b,** Open circuit potential (OCP) measurement on p-InGaN photochemical diode nanostructure arrays under dark and full arc illumination. The potential difference between light and dark is positive, indicating p-type behavior of the photochemical diodes.

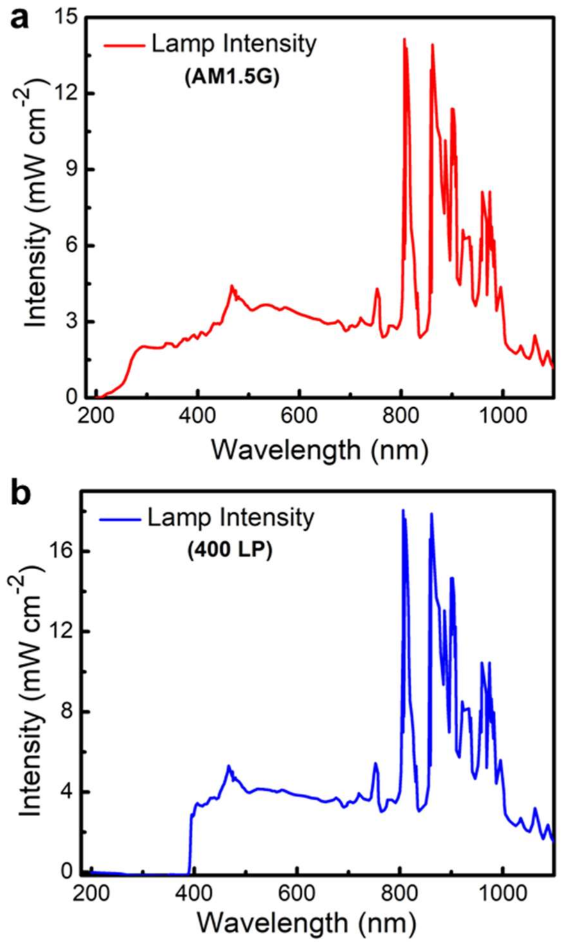

Supplementary Figure 8: Output spectra of the irradiation source. Typical output spectral distribution of 300 W Xenon lamp (onto the sample surface) with **a,** AM1.5 G filter and **b,** 400 nm long-pass filter. The energy conversion efficiency (ECE) and solar to hydrogen efficiency (STH) of the photochemical diode is estimated to be ~17.5% and ~3.3%, respectively, in the spectral range of 200-485 nm and full arc (200-1100 nm), respectively.

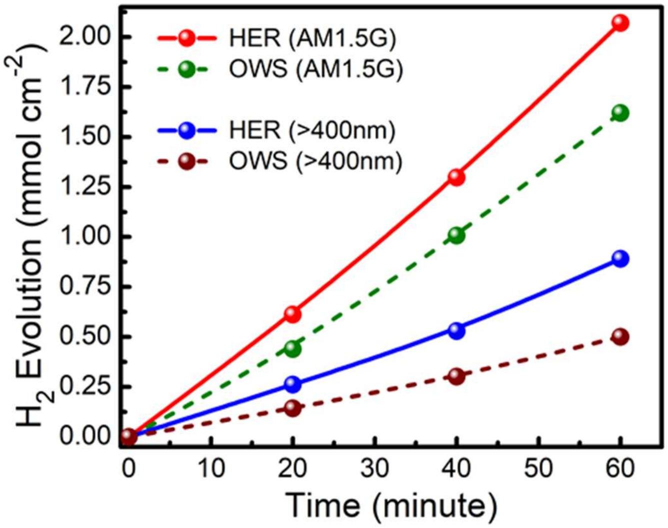

Supplementary Figure 9: Hydrogen evolutions from GaN/InGaN photochemical diode arrays. Comparative hydrogen production as a function of time from the photochemical diode nanosheet arrays in hydrogen evolution reaction using aqueous methanol solution (denoted as HER) and in neutral pH overall water splitting (denoted as OWS), under concentrated excitation of ~32 suns using AM1.5G and 400 nm long pass filters.

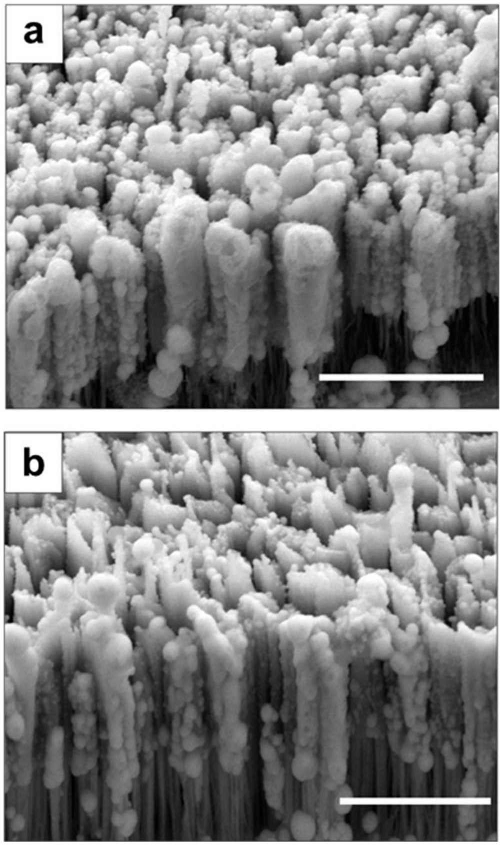

Supplementary Figure 10: Structural stability of the photochemical diodes after reactions. SEM image of the *p*-GaN/*p*-$In_{0.22}Ga_{0.78}N$ photochemical diode photocatalyst after ~4 hours of **a,** overall neutral pH (~7.0) water splitting (OWS) reaction and **b,** hydrogen evolution reaction (HER) in aqueous methanol solution, demonstrating the stability of the nanostructures. Rh/Cr_2O_3 core-shell nanostructures and Rh nanoparticles were deposited as a co-catalyst on the photochemical diode arrays in **a,** and **b,** for OWS and HER, respectively. The stability of the co-catalysts was also checked using TEM analysis and does not show noticeable degradation. Scale bars, 1 μm.

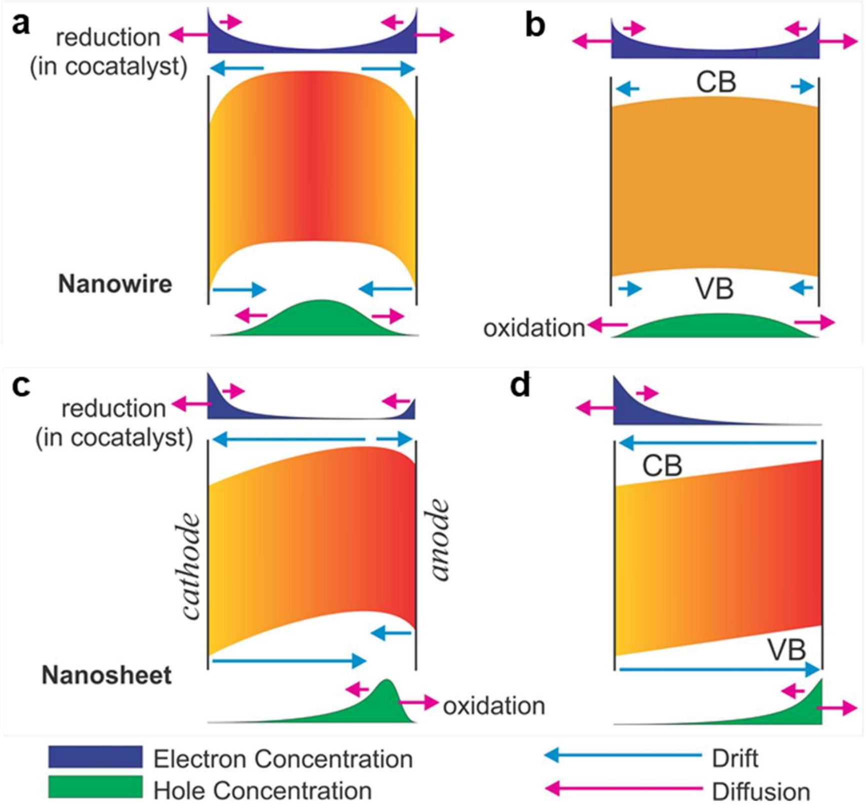

Supplementary Figure 11: Dominant carrier-transport in nanowires and nanosheets. a, Optimally doped nanowire provides carrier separation in near-surface region under low excitation, however, with low photocatalytic activity due to trapping of photo-generated holes in the bulk. **b,** Under concentrated irradiation, reduced band-bending in nanowire lowers the hole-diffusion barrier for water oxidation, the rate-limiting step for overall water splitting. However, probability of recombination also increases due to the overlap of electron and hole concentration in the bulk, thus limiting the photocatalytic efficiency. Carrier transport mechanism in a photochemical diode nanosheet under **c,** low excitation and **d,** concentrated high excitation. Built-in potential and associated band bending due to Mg-doping gradient plays critical role in carrier separation, thereby suppressing charge carrier recombination both inside the bulk and in the near-surface region.

Supplementary Note 1

Conventional photochemical diodes/hetero-structures and their water splitting approaches

Balanced overall water splitting produces one molecule of oxygen and two molecules of hydrogen from two molecules of water ($2H_2O = 2H_2 + O_2$). The Gibbs free energy and enthalpy of the water splitting reaction under ambient pressure and temperature are $\Delta G^0 = 237$ kJ mol^{-1} and $\Delta H^0 = 286$ kJ mol^{-1}, respectively. The energy is converted into the standard cell voltage as $\Delta G^0 = nFE^0$, in which n is the number of electrons, F is Faraday's constant, and E^0 is the standard cell voltage. From the equation, thermodynamically required potential for splitting one molecule of H_2O (to produce one molecule of H_2) is calculated as 1.23 eV, which requires two electrons as active participant in the reaction. Therefore, four active electrons are required for balanced water splitting (for stoichiometric production of H_2 and O_2 with a ratio 2:1).

The photochemical diode offers the advantages of enhanced charge carrier separation within the photon absorber(s) due to built-in electric field and can efficiently steer them towards distinct catalytic surfaces for redox-half reactions. Schematic depiction of a Schottky-type photochemical diode (Supplementary Fig. 1a) shows that the n-type semiconductor performs as a single bandgap absorber, and the photo-generated carriers (holes and electrons) are separated due to built-in electric field, migrates towards semiconductor-electrolyte interface and metal-electrolyte interface for water oxidation and proton reduction reaction, respectively. Schematic of a conventional p-n photochemical diode energy levels for overall water splitting are presented in Supplementary Fig. 1b. The p-n photochemical diode is a minority carrier device, i.e. the minority holes in n-type (photo-anode) and electrons in p-type (photo-cathode) migrates towards semiconductor/electrolyte interface and participates in water oxidation and proton reduction reaction, respectively. Majority carriers, on the other hand, recombine in the ohmic contact. With appropriate band-bending at the

semiconductor-electrolyte interfaces, a conventional p-n photochemical diode offers the advantage of efficient charge carrier separation and the use of single semiconductor with narrow-bandgap or with complementary bandgaps for simultaneous extended absorption of UV and visible light. However, it requires eight photons to produce four active electron-hole pairs due to majority carrier recombination, and hence, for a particular threshold absorption wavelength the maximum efficiency for solar energy conversion get reduced to half compared to that for a single-absorber approach of overall water splitting[1-3]. Besides, for semiconductors with complementary bandgaps, matching in threshold and equal photon absorption wavelength is required for balanced redox reaction in order to prevent back reaction and corrosion[3-5]. Realization of nanoscale ohmic contact at the lateral (p/n) interfaces of vertically aligned photochemical diode is difficult, therefore making it suitable only for planar devices. On the other hand, through junction engineering, it is typically hard to ensure sufficient over-potentials and appropriate band-bending for both oxidation and reduction reactions in overall photocatalytic water splitting (Supplementary Fig. 1c). The p-n junction is an effective approach to individually engineer either oxidation or reduction reaction forward at the nanoscale[6-9]. However, for overall unassisted photocatalytic water splitting, it offers band-bending at the semiconductor-electrolyte interface that hinders the migration of the appropriate carriers (both electrons and holes) to participate in respective half-reactions, resulting in reduced efficiency. To a greater extent, both of these approaches offer internal electric field as a driving force for unidirectional electron and hole transfer only at the near-surface region and near the space-charge region (SCR) at the interface. Moreover, the interfaces (junctions) need to be carefully optimized in order to improve the photocatalytic performance, by tailoring the interfacial parameters, namely, compositions, interfacial area, defects, electronic coupling between surfaces, and the distance from the interface to the active redox sites[10], thus making the implementation

more challenging. If the distance from the interface to the surface is large (thick semiconductor layers), potential difference is required to govern the charge transfer from the semiconductor interface to the bulk or from within the bulk to the reactive surfaces, thus limiting its performance in zero-bias overall water splitting.

Supplementary Note 2

PL intensity and suppressed carrier-recombination in GaN nanosheet photochemical diode

To reveal the origin of reduction in PL intensity for nanosheet photochemical diodes, we further investigated the characteristics of photochemical diode nanostructures compared to axially symmetric nanowires. The photoluminescence (PL) signal strength has often been directly correlated with the charge carrier trapping, their migration and transfer efficiency, and corresponding photocatalytic activity of the device. In numerous studies, the PL intensity of photocatalytic material was found to decrease as a function of noble metal loading. This has been ascribed to the electron-scavenging effect of metal nanoparticles that act as electron acceptor owing to their larger work function[11-13]. Apart from noble metals, PL intensity also gets reduced in general due to the efficient electron and hole capture by hydrogen evolution reaction (HER) co-catalyst (such as WO_3, WSe_2, SnO_2) and oxygen evolution reaction (OER) co-catalyst (such as PbS, MnO_2, Co_3O_4), respectively[14-15]. In our previous studies on III-nitride nanowires, Rh/Cr_2O_3 core-shell structure had been successfully used as efficient HER co-catalyst[16-17] and CoOx had been explored to act as an efficient OER co-catalyst in numerous other reports[18]. The PL intensity from co-catalyst nanoparticles decorated GaN nanowires was found to get reduced by 50-60% (Supplementary Fig. 2a) due to electron and hole trapping by Rh/Cr_2O_3 and Co_3O_4 respectively. Simultaneous deposition of both HER and OER co-catalysts also reduces the PL intensity

further[15,19]. However, as depicted in Supplementary Fig. 2a, six-fold reduction in the PL intensity has been observed for bare GaN photochemical diode compared to that of bare GaN nanowires, grown under similar condition.

Supplementary Note 3

Structural, optical and catalytic properties of GaN photochemical diodes

The prominent reflections of 002 in the X-ray diffraction (XRD) pattern shows negligible broadening or shift for GaN:Mg photochemical diodes compared to that of the nanowires. As shown in Supplementary Fig. 2b, the narrow peak widths of the 002 (and 004, not shown here) reflection further confirms the single crystalline structure of the as-grown Mg-doped GaN photochemical diodes; and the lattice constant extracted from the XRD pattern is c = 0.5186 nm, which is comparable to the wurtzite crystal structure of non-doped and Mg-doped GaN nanowires with the c-axis (0001) aligned along the growth direction[20]. Typical room-temperature Raman spectroscopy of GaN photochemical diodes (see Methods) also unveils spectral response identical to that of optimally Mg-doped GaN nanowire arrays. As depicted in Supplementary Fig. 2c, both p-GaN nanowires and photochemical diodes reveal the pronounced Mg-induced local vibrational modes (LVMs) at 655 cm^{-1}, and decoupling phenomenon of A_1(LO) phonon mode (735 cm^{-1}) from the longitudinal optical phonon-plasmon coupled (LOPC) mode, present at 740 cm^{-1} in non-doped GaN nanowires[21]. The appearance of S_2 mode (709 cm^{-1}) in photochemical diode can be ascribed to the decoupling and enhancement of surface optical (SO) phonon mode due to high surface to volume ratio and higher fill factor, compared to GaN nanowire arrays[22] (not shown here). Besides, 1~2 cm^{-1} shift in E_2^H peak position is also observed in p-GaN, which can be ascribed to the increased tensile strain due to Mg-doping in nanowires, followed by Mg-doping gradient in

photochemical diodes. Room-temperature Raman spectroscopy of p-GaN in the high energy range also reveals four distinct modes at 2129, 2148, 2166 and 2185 cm^{-1}, as shown in Supplementary Fig. 2d, which can be attributed to the LVMs associated with Mg-H complexes or H-decorated nitrogen vacancies[21,23]. With the same Mg cell-temperature and growth condition, one of the sidewalls of GaN photochemical diode becomes highly p-type due to Mg-doping gradient, compared to the uniform dopant distribution on the surface of axially symmetric GaN nanowires. This can potentially contribute to the larger shift in E_2^H peak position (Supplementary Fig. 2c) and enhanced intensity of high-energy modes (Supplementary Fig. 2d). Nearly 75% enhancement in photocatalytic activity of p-GaN photochemical diodes compared to that of the axially symmetric p-GaN nanowires under similar excitation (Supplementary Fig. 3) further confirms the crystalline quality and efficient migration of charge carriers towards the surface of the photochemical diode nanostructures. GaN nanostructures were used for the comparative study instead of InGaN to avoid the ambiguity, such as peak broadening and shifts in XRD and Raman spectroscopy associated with indium-fluctuation in different samples and regions.

Supplementary Note 4

Facet-selective charge separation, co-catalyst deposition and redox reaction

Efficient charge separation through controlled exposure of facets that are either highly reactive or anisotropic, or possess different work function had been well-accepted on various semiconductors, such as TiO_2, $BiVO_4$, Cu_2WS_4, Cu_2O, BiOCl, $Sr_2Nb_2O_7$, $BaTi_4O_7$, $BaLa_4Ti_4O$, WO_3, $BiVO_4$, $PbTiO_3$, Ag_3PO_4 and $SrTiO_3$[24-26]. The enhanced photocatalytic activity derived from these structures though selective distribution of co-catalysts is mostly ascribed to the spatial separation of redox sites and reduced charge carrier recombination. Spatial separation of the co-catalyst

nanoparticles can further reduce the probability of charge carrier recombination and back reaction[27,28]. Synergetic effect of the facet-preferential charge separation and subsequent distinction of active redox sites had been analyzed in numerous studies[29-31] through selective deposition of OER (for oxidation) and HER (for reduction) co-catalysts on the hole- and electron-rich facets, respectively. In situ photo-deposition of noble metals and metal-oxides successfully determines the locations of the respective photo-generated carriers corresponding to the deposition mechanism[32-34]. In our study, further investigation on the surface properties using TEM and XPS reveals that the suppressed recombination, supported by reduced PL intensity can be correlated to the intrinsic separation of the photo-generated charge carriers, which consequently results in electron and hole enrichment on two distant parallel facets of the InGaN photochemical diode.

Supplementary Note 5

Structural properties of double-band GaN/InGaN photochemical diode, and the role of Rh/Cr_2O_3 core-shell co-catalyst

Detailed scanning transmission electron microscopy (STEM) and energy dispersive X-ray scanning (EDXS) analysis was conducted to explore structural and elemental characterization of the as-grown photocatalyst materials and of the co-catalyst nanostructures. STEM secondary electron (SE) image of Rh/Cr_2O_3 cocatalyst-decorated single photochemical diode nanostructure reveals the existence of p-type $In_{0.22}Ga_{0.78}N$/GaN dual-band structure of ~2 μm in length. EDXS elemental mapping of the In, Ga and N components confirms the existence of a continuous long InGaN segment, with Rh and Cr_2O_3 nanoparticle distribution along the nanowire surfaces. Rh co-catalysts are deposited on the electron-enriched surface, both in small crystalline nanoparticle and nanocluster form as shown in Supplementary Fig. 4. Low resolution STEM-SE image and EDXS

mapping of Rh/Cr_2O_3 deposited nanostructure reveals the existence of Rh (core) and Cr_2O_3 (shell) nanoparticles on the surface of the photochemical diode. As explained in our previous studies[16-17], Rh nanoparticles act as proton reduction co-catalysts by scavenging photoexcited electrons and enhancing charge separation on the surface (Supplementary Fig. 2a), and they do not take part in water oxidation independently. This is supported by a more negative Fermi level of Rh (-4.9 eV) compared to the electron affinity of $In_{0.22}Ga_{0.78}N$:Mg (-3.95 eV), and demonstrated by the reduction in PL intensity on Rh-decorated GaN nanowires. Further deposition of Cr_2O_3 shell on Rh nanoparticles prevents the back reaction on Rh nanoparticles (to create water from hydrogen and oxygen) through the formation of a diffusion barrier which is permeable to H^+ and H_2 but not to O_2 [35]. Growth conditions of the photochemical diodes were optimized to reduce the phase segregation or dislocations in $In_{0.22}Ga_{0.78}N$/GaN layers and to demonstrate excellent crystalline quality, which is further confirmed by clear lattice fringes in the high-resolution TEM image (shown in Figs. 2c-2d).

Supplementary Note 6

Photoelectrochemical (PEC) characterization of the photochemical diode nanostructure: p-type behaviour of $In_{0.22}Ga_{0.78}$N:Mg

Photo-electrochemical cell is a very powerful and efficient tool to characterize certain properties of the photocatalytic material. In order to analyze the conductivity behavior and the junction properties, a single band p-InGaN layer was grown on top of n-GaN nanowire templates on Si n(111) substrate. In-Ga eutectic alloy metal was deposited on the backside of the Si substrate to serve as an Ohmic contact. The measurements were performed by using a 300 W Xenon lamp as an irradiation source with an intensity ~160 mW cm^{-2} on the sample surface. The reactor chamber

was made of quartz to ensure ample transmittance for both UV and visible light. Impedance spectroscopy was performed to acquire the Mott-Schottky (M-S) plot in the dark. A p-n junction characteristic was observed in the M-S plot where a 'V-shape' was present as shown in Supplementary Fig. 7a. A dominant photo-cathodic performance of the photoelectrode can also be seen by the steep negative slope in the M-S plot, which is well supported by the onset potential and negative current in the dark. The shift in the onset potential for photocurrent generation, as depicted in Supplementary Fig. 7a, reflects the catalytic activity of the photoelectrode. It is important to note that these samples were not optimized for photoelectrochemical water splitting studies, given that p-InGaN is grown directly on an n-type template/substrate.

The Mg-doped InGaN nanostructures were further characterized by open circuit potential (OCP) measurement in 1 mol L^{-1} HBr using three-electrode electrochemical cell configuration. The nanostructure arrays (on Si substrate) were used as the photoactive working electrode, whereas a Pt wire, and a double-junction Ag/AgCl were used as counter electrode and reference electrode, respectively. While the nanostructure shows p-n junction characteristics, dominant conductivity type of the material in the electrolyte can be determined by the sign of OCP shift upon illumination. For p-type material, the OCP will be shifted towards more positive potential with respect to Ag/AgCl upon illumination. As shown in Supplementary Fig. 7b, the positive potential difference (with respect to Ag/AgCl) between light and dark under full arc illumination indicates p-type behavior of the nanostructures. The weak n-type behavior in the M-S plot and OCP measurement can be ascribed to the contribution from GaN:Ge template on the Si substrate (see Methods).

Supplementary Note 7

Efficiency of photocatalytic solar water splitting

Herein, we define some of the most common efficiency terms that are thoroughly used in the evaluation of device performance in photocatalytic water splitting. The apparent quantum efficiency (AQE) for the photocatalytic overall water splitting is derived by calculating the number of high energy (above-bandgap) photons corresponding to the incident power. The incident power on the nanostructured photocatalyst and the substrate can be expressed as:

$$P_{\text{incident}}(\lambda) = \rho_{\text{incident}}(\lambda) \times A_{\text{substrate}} \times \beta_{\text{ff}} \quad (1)$$

where $A_{\text{substrate}}$ is the area of the substrate on which the nanostructured photocatalysts are standing, β_{ff} is the nanostructure fill factor, $\rho_{\text{incident}}(\lambda)$ is the incident power intensity on the substrate, which can be derived by measurements using a broadband detector. The β_{ff} is considered as unity (assuming all the incident photons are absorbed) for the calculation of AQE. The number of incident photons per second, as a function of wavelength is calculated from,

$$N_{\text{ph}}(\lambda) = \frac{P_{\text{incident}}(\lambda)}{E_{\text{ph}}(\lambda)} \quad (2)$$

where $E_{\text{ph}}(\lambda) = \frac{hc}{\lambda}$ is the photon energy for the corresponding wavelength. The total number of incident photons per second within $(\lambda_1 - \lambda_2)$ wavelength range can be calculated as follows:

$$N_{\text{ph,inc}}(\lambda_1 - \lambda_2) = \int_{\lambda_1}^{\lambda_2} \frac{P_{\text{incident}}(\lambda) \times \lambda}{hc} d\lambda \quad (3)$$

The AQE is then derived from the following equation:

$$\text{AQE} = 2 \times \frac{\text{Number of evolved } H_2 \text{ molecules per hour}}{\text{Number of incident photons per hour}} \times 100\ \% \quad (4)$$

If we neglect the light trapping and scattering effect in the photocatalyst nanostructure, the APCE can be estimated from equation (4) by replacing the number of incident photons with that of the absorbed photons, derived as:

$$N_{\text{ph,abs}}(\lambda_1 - \lambda_2) = \int_{\lambda_1}^{\lambda_2} \frac{P_{\text{absorbed}}(\lambda)\times\lambda}{hc} d\lambda \quad (5)$$

$P_{\text{absorbed}}(\lambda)$ can be calculated from the power incident on the sample and depends on the absorption co-efficient and threshold wavelength associated with the bandgap of the material, and on the morphology and dimension of the nanostructured photocatalyst. Clearly, this APCE of the nanowire structure is only limited by the charge transport efficiency to the solid-liquid interface ($\eta_{\text{transport}}$) and the efficiency of interfacial charge transfer ($\eta_{\text{interface}}$).

Energy conversion efficiency (ECE) is defined as the ratio of the storable chemical energy (from photocatalysis) to the absorbable photonic energy available to the photocatalyst on the substrate (limited by the absorption threshold), and is calculated as

$$\text{ECE} = \frac{H_2\left(\frac{\text{mmol}}{\text{s}}\right)\times 237\ (\text{kJ})\ \times 1000}{\rho_{\text{incident\_total}}\ (\lambda_1-\lambda_2)\left(\frac{\text{mW}}{\text{sq.cm}}\right)\times A_{\text{substrate}}(\text{sq.cm})} \times 100\ \% \quad (6)$$

Solar-to-hydrogen (STH) conversion efficiency is the most common platform for comparing how efficiently a photocatalyst material can utilize the energy available from the entire solar spectrum. For one-sun (non-concentrated) irradiation (100 mW cm^{-2}) at room temperature and atmospheric pressure, STH can be calculated as

$$\text{STH} = \frac{H_2\left(\frac{\text{mmol}}{\text{s}}\right)\times 237\ (\text{kJ})\ \times 1000}{100\left(\frac{\text{mW}}{\text{sq.cm}}\right)\times A_{\text{substrate}}(\text{sq.cm})} \times 100\ \% \quad (7)$$

Supplementary Note 8

Estimation of AQE, ECE and STH of the double-band p-GaN/p-InGaN photochemical diode

The refractive index mismatch between GaN and InGaN (for In composition ~20-25%) is negligible, and hence the light propagation and absorption inside a layered structure can generally be calculated by using a multi-layer reflectivity model. The reflection from GaN/InGaN interfaces can be ignored for both UV (average reflection~0.05%) and visible light (average reflection~0.005%). In the double-band nanowire heterostructure, GaN absorption spectrum largely excludes visible light whereas InGaN can absorb both UV and visible spectrum[17].

To derive the AQE for the photocatalytic overall water splitting, the number of incident photons in the wavelength range of 200-485 nm (PL peak at ~485 nm as shown in Fig. 4a) was estimated from the lamp. The AQE was then derived using the following process. The incident power on the sample can be calculated using equation (1), where $A_{\mathrm{substrate}}$ is the sample area (~ 3.0 cm^2) and β_{ff} is the nanowire fill factor (unity). $\rho_{\mathrm{incident}}(\lambda)$ is the incident power intensity on the sample, which was derived by measurements using a broadband detector (Thermopile Sensor, Newport-818P-100-55) and considering the power spectrum of the lamp (as shown in Supplementary Fig. 8) and optical filter, while mimicking the experimental configuration.

Calculation of AQE using AM1.5G filter in the UV-visible wavelength range (200-485 nm)

In what follows, we first describe the calculation of the AQE with the use of AM1.5G optical filter. The total incident power on the sample is estimated to be 611 mW cm^{-2} in the wavelength range of 200-485 nm with AM1.5G filter. The number of incident photons per second is calculated from equation (2) as a function of wavelength. The total number of incident photons per second within 200-485 nm wavelength range, and corresponding AQE can be calculated from equation (3) and (4). Considering the entire absorption spectrum (200-485 nm) for the double-band structure, which

includes both UV and part of the visible photons, the AQE was derived to be ~45.85% for the photochemical diode nanostructures, compared to ~20% for the axially symmetric double-band nanowires on Si substrate.

Calculation of the AQE in the visible wavelength range (400-485 nm)

The total incident power intensity (on the sample surface) in the visible wavelength range (400-485 nm) were calculated to be 376 mW cm^{-2}, which corresponds to an AQE of ~20% and ~12% for the photochemical diode and the nanowire structure, respectively.

Calculation of ECE and STH using AM1.5G filter

The ECE was calculated from equation (6)[21], and found to be ~17.5% for the photochemical diode, compared to ~7.5% for the nanowire counterpart in the wavelength range of (200-485 nm). On the other hand, in the wavelength range of 400-485 nm, the calculated values for ECE were ~8.75% and 5.3%, respectively. STH efficiency was calculated for both the photocatalyst nanostructures in the wavelength range 200-1100 nm using AM1.5G filter. The STH was estimated to be ~3.3% and 1.5% for the photochemical diodes and nanowire structures, respectively. The effect of pressure and temperature on STH-estimation was neglected herein to avoid ambiguity. For example, considering the initial low-pressure of the reaction chamber (~10 kPa) and final high-temperature of water (~318-325 K), the Gibbs energy of water splitting reaction can be as low as ~231 kJ mol^{-1}. However, the water temperature is low at the beginning (~293 K), and the chamber pressure increases gradually as the reaction proceeds forward under concentrated irradiation (Supplementary Movie 1 and 2). Therefore, the Gibbs energy of water splitting reaction can vary within ~231-237 kJ mol^{-1}, and the STH can be estimated in the range of ~3.21-3.3%, respectively, which lies almost within experimental error.

Efficiency of the photochemical diode in aqueous methanol solution

The photocatalytic activity of Mg-doped $In_{0.22}Ga_{0.78}N$/GaN nanostructures in HER inside aqueous CH_3OH solution (5:1 water/alcohol ratio) provided H_2 evolution rate of ~2.07 mmol $h^{-1}cm^{-2}$ under full arc illumination using AM1.5G filter. Corresponding apparent quantum efficiency (AQE) was estimated to be ~58.6% for an incident intensity of ~611 mW cm^{-2} in the wavelength range of 200-485 nm. Supplementary Fig. 9 shows the comparative hydrogen evolution per unit area (substrate) for different excitation and experimental conditions. Under visible light irradiation using a 400 nm long pass filter, for an incident intensity of ~376 mW cm^{-2} in the wavelength range of 400-485 nm, the H_2 evolution rate was measured ~0.89 mmol $h^{-1}cm^{-2}$ with an estimated AQE of ~35.5% for HER.

Supplementary Note 9

Carrier transport in photochemical diode nanosheet: Impact on efficiency transformation

It is important to realize that, the photocatalytic activity of a semiconductor is greatly affected by the dynamic behaviors of photo-generated charge carriers. The separation/migration and the recombination process of the photo-generated electron-hole pairs are two competitive pathways. The overall water splitting becomes most efficient when the bulk-separation and surface-trapping of the charge carriers surpasses their recombination while retarding the latter one. MBE grown non-doped GaN/InGaN nanowires are weakly n-type, which, together with surface depletion effect, creates a high degree of upward band-bending in near-surface region and suppresses electron transfer to the adsorbates. With n-type dopant incorporation (Si/Ge), the upward band-bending of the nanostructures becomes more severe, providing little or no control. On the contrary, band-bending and surface properties can be optimized in Mg-doped p-type GaN/InGaN nanowires

to provide significantly enhanced photocatalytic efficiency, while simultaneously driving the water oxidation and the proton reduction reactions forward and minimizing defects in the nanostructures[16,17,36]. The decoration of nanowire surface with reduction co-catalysts (HER), in addition, accelerates carrier separation from near-surface region. Bulk recombination, however, persists due to its flat-band nature, which results in trapping of photo-generated holes in the bulk of nanowires. Owing to the barrier for directional (radial) migration towards the non-polar surface, these trapped holes cannot participate in the surface reaction and hence diffuse randomly[36] with higher probability of recombination. Under concentrated excitation, the band bending is reduced which lowers the barrier for hole diffusion with enhanced water oxidation reaction, however, not to its full potential. One of the reasons is that, the random distribution of HER-cocatalyst on nanowire surface greatly limits available surface area for water oxidation, which is a 4-electron sluggish process, and the efficiency of overall water splitting depends on effective rate of water oxidation. Even though the transport is no longer diffusion limited, under flat-band condition the concentration-gradient for holes in valence band is reduced. This leads to overlapping of electron and hole distribution with enhanced probability of recombination (Supplementary Fig. 11a-b).

The photochemical diode nanosheet breaks the bottleneck of charge carrier separation by electric-field driven drift-dominated carrier transport. While the band bending in a photochemical diode is favorable for carrier separation towards catalytic surfaces (cathode and anode), it also largely prevents reverse diffusion current (Supplementary Fig. 11c-d) against electric-filed induced barrier, thereby making the carrier-flow unidirectional. HER co-catalysts on the cathode surface further eliminate the gradient in electron concentration (conduction band) and associated reverse diffusion current, by trapping the accumulated electrons from near-surface region to accelerate proton reduction reaction. Critically important is that, electrons and holes in a

photochemical diode can freely diffuse along the non-polar surfaces (parallel cathode and anode) towards cocatalyst-enriched or reaction-prone regions, respectively, with the least probability of interference, crowding, and recombination. This leads to further enhancement in charge transport efficiency to the solid-liquid interface ($\eta_{\text{transport}}$) and the efficiency of interfacial charge transfer ($\eta_{\text{interface}}$). Consequently, impressive solar-to-hydrogen conversion efficiency can be derived from the photochemical diode nanosheet.

SUPPLEMENTARY REFERENCES

1 Wang, Q. *et al.* Scalable water splitting on particulate photocatalyst sheets with a solar-to-hydrogen energy conversion efficiency exceeding 1%. *Nat. Mater.* **15**, 611-615 (2016).

2 Yeh, T.-F., Teng, C.-Y., Chen, S.-J. & Teng, H. Nitrogen-Doped Graphene Oxide Quantum Dots as Photocatalysts for Overall Water-Splitting under Visible Light Illumination. *Adv. Mater.* **26**, 3297-3303 (2014).

3 Bolton, J. R., Strickler, S. J. & Connolly, J. S. Limiting and realizable efficiencies of solar photolysis of water. *Nature* **316**, 495-500 (1985).

4 Blankenship, R. E. *et al.* Comparing Photosynthetic and Photovoltaic Efficiencies and Recognizing the Potential for Improvement. *Science* **332**, 805 (2011).

5 Doscher, H., Geisz, J. F., Deutsch, T. G. & Turner, J. A. Sunlight absorption in water - efficiency and design implications for photoelectrochemical devices. *Energy Environ. Sci.* **7**, 2951-2956 (2014).

6 Nozik, A. J. p-n photoelectrolysis cells. *Appl. Phys. Lett.* **29**, 150-153 (1976).

7 Meng, F., Li, J., Cushing, S. K., Zhi, M. & Wu, N. Solar Hydrogen Generation by Nanoscale p–n Junction of p-type Molybdenum Disulfide/n-type Nitrogen-Doped Reduced Graphene Oxide. *J. Am. Chem. Soc.* **135**, 10286-10289 (2013).

8 Zhang, K., Kim, W., Ma, M., Shi, X. & Park, J. H. Tuning the charge transfer route by p-n junction catalysts embedded with CdS nanorods for simultaneous efficient hydrogen and oxygen evolution. *J. Mater. Chem. A* **3**, 4803-4810 (2015).

9 Ahmed Zelekew, O. & Kuo, D.-H. A two-oxide nanodiode system made of double-layered p-type Ag_2O@n-type TiO_2 for rapid reduction of 4-nitrophenol. *Phys. Chem. Chem. Phys.* **18**, 4405-4414 (2016).

10 Liu, C., Tang, J., Chen, H. M., Liu, B. & Yang, P. A Fully Integrated Nanosystem of Semiconductor Nanowires for Direct Solar Water Splitting. *Nano Lett.* **13**, 2989-2992 (2013).

11 Liqiang, J. *et al.* Review of photoluminescence performance of nano-sized semiconductor materials and its relationships with photocatalytic activity. *Sol. Energy Mater Sol. Cells* **90**, 1773-1787 (2006).

12 Chen, S. F. *et al.* Large scale photochemical synthesis of M@TiO_2 nanocomposites (M = Ag, Pd, Au, Pt) and their optical properties, CO oxidation performance, and antibacterial effect. *Nano Res.* **3**, 244-255 (2010).

13 Bumajdad, A. & Madkour, M. Understanding the superior photocatalytic activity of noble metals modified titania under UV and visible light irradiation. *Phys. Chem. Chem. Phys.* **16**, 7146-7158 (2014).

14 Lin, Z. *et al.* A Floating Sheet for Efficient Photocatalytic Water Splitting. *Adv. Energy Mater.* **6**, 1600510 (2016).

15 Yang, J., Wang, D., Han, H. & Li, C. Roles of Cocatalysts in Photocatalysis and Photoelectrocatalysis. *Acc. Chem. Res.* **46**, 1900-1909 (2013).

16 Kibria, M. G. *et al.* Tuning the surface Fermi level on p-type gallium nitride nanowires for efficient overall water splitting. *Nat. Commun.* **5**, 3825 (2014).

17 Kibria, M. G. *et al.* Visible light-driven efficient overall water splitting using p-type metal-nitride nanowire arrays. *Nat. Commun.* **6**, 6797 (2015).

18 Zhong, M. *et al.* Surface Modification of CoOx Loaded $BiVO_4$ Photoanodes with Ultrathin p-Type NiO Layers for Improved Solar Water Oxidation. *J. Am. Chem. Soc* **137**, 5053-5060 (2015).

19 Li, A. *et al.* Spatial separation of oxidation and reduction co-catalysts for efficient charge separation: Pt@TiO_2@MnOx hollow spheres for photocatalytic reactions. *Chem. Sci.* **7**, 890-895 (2016).

20 Kibria, M. G. *et al.* Atomic-Scale Origin of Long-Term Stability and High Performance of p-GaN Nanowire Arrays for Photocatalytic Overall Pure Water Splitting. *Adv. Mater.* **28**, 8388-8397 (2016).

21 Wang, Q. *et al.* p-Type dopant incorporation and surface charge properties of catalyst-free GaN nanowires revealed by micro-Raman scattering and X-ray photoelectron spectroscopy. *Nanoscale* **6**, 9970-9976 (2014).

22 Wang, J. *et al.* Anticrossing of axial and planar surface-related phonon modes in Raman spectra of self-assembled GaN nanowires. *Phys. Rev. B* **85**, 155432 (2012).

23 Kaschner, A. *et al.* Local vibrational modes in Mg-doped GaN grown by molecular beam epitaxy. *Appl. Phys. Lett.* **74**, 3281-3283 (1999).

24 Moniz, S. J. A., Shevlin, S. A., Martin, D. J., Guo, Z.-X. & Tang, J. Visible-light driven heterojunction photocatalysts for water splitting - a critical review. *Energy Environ. Sci.* **8**, 731-759 (2015).

25 Kudo, A. & Miseki, Y. Heterogeneous photocatalyst materials for water splitting. *Chem. Soc. Rev.* **38**, 253-278 (2009).

26 Osterloh, F. E. Inorganic Materials as Catalysts for Photochemical Splitting of Water. *Chem. Mater.* **20**, 35-54 (2008).

27 Li, R., Tao, X., Chen, R., Fan, F. & Li, C. Synergetic Effect of Dual Co-catalysts on the Activity of p-Type Cu_2O Crystals with Anisotropic Facets. *Chem. Eur. J.* **21**, 14337-14341 (2015).

28 Wang, D. *et al.* Core/Shell Photocatalyst with Spatially Separated Co-Catalysts for Efficient Reduction and Oxidation of Water. *Angew. Chem. Int.* **52**, 11252-11256 (2013).

29 Zhang, Q. *et al.* The dependence of photocatalytic activity on the selective and nonselective deposition of noble metal cocatalysts on the facets of rutile TiO_2. *J. Catal.* **337**, 36-44 (2016).

30 Min, S., Wang, F. & Lu, G. Graphene-induced spatial charge separation for selective water splitting over TiO_2 photocatalyst. *Catal. Commun.* **80**, 28-32 (2016).

31 Li, R. *et al.* Spatial separation of photogenerated electrons and holes among {010} and {110} crystal facets of $BiVO_4$. *Nat Commun.* **4**, 1432 (2013).

32 Mu, L. *et al.* Enhancing charge separation on high symmetry $SrTiO_3$ exposed with anisotropic facets for photocatalytic water splitting. *Energy Environ. Sci.* **9**, 2463-2469 (2016).

33 Zhang, Q. *et al.* Effect of Redox Cocatalysts Location on Photocatalytic Overall Water Splitting over Cubic $NaTaO_3$ Semiconductor Crystals Exposed with Equivalent Facets. *ACS Catal.* **6**, 2182-2191 (2016).

34 Li, R., Han, H., Zhang, F., Wang, D. & Li, C. Highly efficient photocatalysts constructed by rational assembly of dual-cocatalysts separately on different facets of $BiVO_4$. *Energy Environ. Sci.* **7**, 1369-1376 (2014).

35 Maeda, K. & Domen, K. Photocatalytic Water Splitting: Recent Progress and Future Challenges. *J. Phys. Chem. Lett.* **1**, 2655-2661 (2010).

36 Chowdhury, F. A., Mi, Z., Kibria, M. G. & Trudeau, M. L. Group III-nitride nanowire structures for photocatalytic hydrogen evolution under visible light irradiation. *APL Mater.* **3**, 104408 (2015).
