# Supplementary material for: A photochemical diode artificial photosynthesis system for unassisted high efficiency overall pure water splitting
Source: Nat Commun. 2018 Apr 27;9:1707. doi: 10.1038/s41467-018-04067-1 (PMC5923260; doi:10.1038/s41467-018-04067-1)
Supplement: Supplementary file 2 — Description of Additional Supplementary Information [file 41467_2018_4067_MOESM2_ESM.pdf]

Description of Additional Supplementary Files

File Name: Supplementary Movie 1
Description: Spontaneous and steady-state photocatalytic overall neutral pH water splitting in real time, showing clear bubble formation at high rate under concentrated irradiation. The light is incident at 30-degree angle from the substrate normal. The year on the coin is 2009.

File Name: Supplementary Movie 2
Description: Photocatalytic overall neutral pH water splitting reaction conducted at temperature slightly below room-temperature (at 18 °C) to clearly probe the bubble formation due to generation of hydrogen and oxygen gases.
